# Assessment of selected interleukins (IL-6, IL-17A, IL-18, IL-23) and chemokines (RANTES, IP-10) in children with acute and chronic urticaria

**DOI:** 10.1186/s13052-022-01395-3

**Published:** 2022-12-21

**Authors:** Anna Góra, Maciej Przybył, Elżbieta Świętochowska, Edyta Machura

**Affiliations:** 1 Department of Paediatrics, Faculty of Medical Sciences in Zabrze, Medical University of Silesia, Katowice, Poland; 2 Clinical Hospital No. 1 in Zabrze, 13-15 3 Maja St, 41-800 Zabrze, Poland; 3 Department of Medical and Molecular Biology, Faculty of Medical Sciences in Zabrze, Medical University of Silesia, Katowice, Poland

**Keywords:** Chemokines, Children, Cytokines, Interleukin-6, Urticaria

## Abstract

**Background:**

Urticarial lesions develop as a result of the activation of mast cells which, through the release of mediators, influence the formation of local inflammatory infiltrates. Changes in the expression of many cytokines and chemokines are observed in the course of urticaria.

The aim of the study was to evaluate serum levels of interleukin (IL)-6, IL-17A, IL-18, IL-23, regulated on activation, normal T cell expressed and secreted (RANTES) and interferon (IFN)-γ-inducible protein-10 (IP-10) in children with acute urticaria and exacerbation of chronic urticaria in comparison to healthy volunteers. Moreover, we made an attempt to identify factors associated with the acute phase of urticaria and factors predicting the course of the disease among the studied parameters.

**Methods:**

We retrospectively analyzed 32 children with acute urticaria and 32 children with chronic urticaria. The control group consisted of 40 healthy children. Each patient was clinically evaluated. Serum concentrations of selected cytokines and chemokines were determined by using enzyme-linked immunosorbent assay.

**Results:**

Patients with acute and chronic urticaria had higher concentrations of IL-6 and IL-17A (*p* < 0.001) and lower concentrations of IL-18, IL-23, RANTES and IP-10 (*p* < 0.001) as compared to the control group. A significant association between IL-6 and IP-10 with the acute phase of urticaria has been demonstrated. There was no correlation of the studied cytokines and chemokines with disease activity.

**Conclusions:**

In children with acute phase of urticaria, the cytokine serum concentration differs compared to healthy subjects. IL-6 and IP-10 seem to be useful in differentiating children with acute phase of urticaria and healthy ones. The search for factors predicting the course of the disease requires further studies.

## Background

Urticaria is a common pediatric dermatosis. It is characterized by the development of wheals and/or angioedema. Acute urticaria (AU) lasts less than 6 weeks and is most often associated with infectious, less commonly allergic or drug-induced factors. In chronic urticaria (CU), skin lesions persist for more than 6 weeks. CU is classified as spontaneous (CSU) and inducible (CIndU) [1]. In approximately 40% of patients, CSU may be associated with autoimmune mechanisms, but the cause often remains unknown [2]. CIndU is associated with a known trigger (e.g., cold urticaria, solar urticaria, contact urticaria) [1]. The development of urticaria is dependent on mast cell activation and degranulation. Histamine and other mediators released by mast cells alter the permeability of the vascular endothelium, which promotes the formation of local inflammatory infiltrates composed mainly of Th (T helper) lymphocytes, granulocytes, and other immune cells [3].

The pathogenesis of chronic urticaria is related to immune mechanisms, the presence of systemic inflammation, and activation of the coagulation and fibrinolysis systems [1, 3]. Increased expression of many cytokines and chemokines is observed in patients with urticaria [4]. It is recognized that an imbalance between cellular (Th1) and humoral (Th2) responses and the activation of a Th17-type response leads to the development and maintenance of urticarial lesions [4].

Interleukin-6 (IL-6) is a multidirectional proinflammatory cytokine that regulates Th1, Th2, and Th17 responses [3]. It exhibits activity against B, T, and NK cells [5], induces mast cell proliferation and chemotaxis [3], and stimulates CRP and fibrinogen synthesis [3]. It is a frequently used marker of inflammation [6]. IL-17A (a member of the IL-17 family) stimulates the production of proinflammatory cytokines, including IL-6 [7]. Together with IL-17, IL-23 forms a common axis coordinating the action of congenital and acquired immune responses [8]. Alterations in IL-17 and IL-23 expression in patients with urticaria contribute to a disturbed pattern of Th17 lymphocyte activity, which is also observed in other autoimmune and allergic diseases such as psoriasis and atopic dermatitis [4, 9]. IL-18 plays an important role in the congenital and acquired immune response [10]. It influences the development of allergic inflammatory response by recruiting neutrophils, increasing histamine release and immunoglobulin E (IgE) production [11].

Chemokines are a group of proteins that regulate the migration of leukocytes from vessels to sites of inflammation or injury, which is crucial for the formation of urticarial lesions [12]. They affect angiogenesis, differentiation and survival of immune cells and hematopoietic stem cells [13, 14]. They are an indicator of endothelial dysfunction present in urticaria [15]. RANTES (Regulated upon Activation, Normal T-cell Expressed and Secreted/CCL5) and IP-10 (interferon-γ-inducible protein-10/CXCL10) affect the recruitment of T-lymphocytes, monocytes and other immune cells [14, 15].

The aim of this study was to evaluate serum levels of the cytokines IL-6, IL-17A, IL-18, IL-23, and the chemokines IP-10 and RANTES in a group of children with acute urticaria and exacerbations of chronic urticaria compared to healthy volunteers. Additionally, an attempt was made to identify potential factors associated with the acute phase of urticaria and predictors of the course of urticaria among the cytokines studied.

## Materials and methods

### Study design and participants

We enrolled 64 children (32 with AU and 32 with CU) aged from 2 to 17 years. The diagnosis of AU and CU was based on the EAACI/GA^2^LEN/EuroGuiDerm/APAAACI guidelines [1]. Patients were hospitalized in the General Pediatric Ward of the Clinical Hospital No. 1 in Zabrze between 2013 and 2019. On admission, history and physical examination were performed. At the same time material was collected for laboratory determinations. The activity of urticaria was evaluated according to the TSS (Total Symptom Score) [16] which is defined as the sum of the scores for the number and size of wheals and severity of pruritus. The maximum diameter of the largest wheal was assessed according to the following scheme: 0 = 0, 1 = diameter ≤ 1.5 cm, 2 = diameter > 1.5 cm and ≤ 2.5 cm, 3 = diameter > 2.5 cm. The number of wheals was estimated consecutively as: 0 = no wheals, 1 = ≤10 wheals, 2 = > 10 wheals, 3 = body covered with wheals. Severity of pruritus was scored as: 0 = absent, 1 = mild, 2 = moderate pruritus with slight disturbance of daily activities and/or sleep, 3 = intense itching with marked disturbance of daily activities and/or sleep. Exclusion criteria included serious general condition, anaphylaxis, hematopoietic or immune diseases, genetic syndromes, and use of antihistamines or systemic glucocorticosteroids during the 7 days preceding hospitalization.

The control group consisted of 40 healthy children matched for sex and age distribution. Children included into the control group attended the outpatient pediatric clinic for routine health checks.

### Laboratory methods

Sera were stored at − 40 °C until assays. Enzyme-linked immunosorbent assay (ELISA) kits were used to measure the levels of cytokines. Serum concentrations of IL-6, IL-17A, IL-23, and IP-10 were measured by the use of Diaclone (France) kits. The sensitivity was respectively: 0.81 pg/ml; 2.3 pg/ml; < 20 pg/ml and 5.7 pg/ml. Absorbance readings were performed using a SYNERGY/H1 (BioTek, USA). IL-18 and RANTES concentrations were determined by the use of Cloud-Clone Corp. (USA) kits with sensitivities of, respectively: 5.6 pg/ml and 0.061 ng/ml. Absorbance readings were performed using a μQuant (BioTek, USA). The obtained results were processed using Gen5v3.05 program (BioTek, USA). The procedures were carried out according to the instructions attached to the kits.

### Statistical evaluation

Statistical analysis was performed using Statistica 13.1, Dell Inc. Descriptive analysis was presented as median and quartiles (Q_25_-Q_75_). The Kruskall-Wallis test was used to compare parameters between groups. The normality of distribution of individual parameters was assessed with the Shapiro-Wilk test. Logistic regression analysis including odds ratio estimation and 95% confidence interval was performed to determine factors associated with the acute phase of urticaria. A Receiver Operating Curve (ROC) was established to evaluate the sensitivity and specificity of the studied parameters. Correlations between selected parameters were evaluated using Spearman’s rank coefficient. *p* values < 0.05 were considered statistically significant.

## Results

There were no significant differences in age, gender, and BMI between the study group and control group. Demographic and clinical characteristics and selected laboratory findings in the study and control groups are shown in Table 1.

**Table 1:** The demographics, clinical characteristic and laboratory findings of urticaria patients compared to the control group

Group/Parameter		Acute urticaria (*n* = 32)	Chronic urticaria (*n* = 32)	Control group (*n* = 40)
Median age (yr) (Q_25_ – Q_75_)		9.21 (7.04–12.13)	11.21 (8–14.42)	11 (7–14)
Range (yr)		2–17	2–17	3–17
Gender (M/F)		17/15	13/19	21/19
BMI (kg/m^2^) (Q_25_ – Q_75_)		17.85 (15.74–21.84)	18.39 (15.99–21.67)	17.35 (16.15–19.88)
Median duration of hospitalization (days) (Q_25_ – Q_75_)		5 (4–6)	4 (3–5)	Not applicable (NA)
Localization of skin changes, n(%)				
- whole body		14 (43.8)	6 (18.8)	
- limbs		6 (18.8)	8 (25)	
- trunck		3 (9.4)	7 (21.9)	
- trunck and limbs		4 (12.5)	8 (25.0)	
- face		5 (15.6)	3 (9.4)	
Edema of the limbs/arthritis, n(%)		15 (46.9)	2 (6.3)	
Angioedema, n(%)		9 (28.1)	5 (15.6)	
Fever > 38 °C, n(%)		10 (31.2)	0	
Abdominal pain, n(%)		4 (12.5)	0	
Wheezing, n(%)		3 (9.4)	1 (3.1)	
Respiratory tract infection, n(%)		21 (65.6)	4 (12.5)	
Other infections, n(%)		3 (9.4)	3 (9.4)	
Parasitic infection, n(%)		3 (9.4)	3 (9.4)	
Allergic disease, n(%)^a^		10 (31.2)	9 (28.1)	
Familial atopy, n(%)		3 (9.4)	13 (40.6)	
Antibiotic therapy, n(%)		6 (18.8)	0	
Use of NSAIDs, n(%)		2 (6.3)	1 (3.1)	
Unknown cause, n(%)		7 (21.9)	14 (43.8)	
Concomitant diseases, n(%)		21 (65.6)	17 (53.1)	
- asthma		1 (3.1)	3 (9.4)	
- atopic dermatitits		2 (6.3)	4 (12.5)	
- allergic rhinitis		3 (9.4)	2 (6.3)	
- chronic tonsillitis/tonsillar hyperthrophy		15 (46.9)	8 (25.0)	
Severity score TSS, n (%)				
Mild (0–3 points)		3 (9.4)	14 (43.8)	
Moderate (4–6 points)		16 (50.0)	8 (25.0)	
Severe (7–9 points)		13 (40.6)	10 (31.2)	
Medians of laboratory data (Q_25_ – Q_75_):				
Hemoglobin (g/dL)		12.76 (12.05–13.4)**	13.63 (12.9–14.51) *	12.88 (12.21–13.81)
Platelets (× 10^3^/uL)		348.5 (273.0–440.0)*	327.0 (271.0–377.5)*	274.5 (223–321)
White blood cells (× 10^3^/uL)		11.18 (7.6–15.63)*/**	7.21 (5.9–9.24)	6.65 (5.59–7.92)
Neutrophil (× 10^3^/uL)		6.44 (3.94–10.95)*/**	3.5 (2.72–5.37)	3.25 (2.31–3.84)
Lymphocyte (× 10^3^/uL)		2.8 (2.13–3.54)	2.48 (2.1–3.38)	2.22 (1.85–3.04)
Neutrophil-to-lymphocyte ratio (NLR)		2.08 (1.69–3.35)*/**	1.33 (0.87–2.2)	1.22 (0.98–1.87)
Platelet-to-lymphocyte ratio (PLR)		125.28 (81.58–160.45)	119.56 (94.97–153.02)	118.87 (84.81–145.27)
C-reactive protein (mg/l)		7.66 (1.02–17.5)*	1.14 (0.55–6.44)*	0.6 (0.34–1.09)
Total IgE (IU/ml)		74.21 (44.21–171.1)	74.41 (26.46–191.55)	33.87 (20.21–89.9)
D-dimer (ng/ml)		1410.0 (690.0–2390.0)**	270.0 (260.0–390.0)	Not applicable (NA)

*NSAIDs* nonsteroidal anti-inflammatory drugs, *TSS* Total Symptom Score

*p < 0.05 in comparison to the control group

**p < 0.05 acute urticaria vs chronic urticaria

^a^positive specific IgE/skin prick tests, positive anamnesis of food and inhalation allergies

In the AU and CU group, regardless of disease severity, significantly higher IL-6 and IL-17A values (*p* < 0.001) and significantly lower IL-18, IL-23, RANTES and IP-10 values (*p* < 0.001) were observed with respect to the control group (CG). Lower RANTES values were also noted in patients with moderate CU compared to severe CU (*p* =  0.02).

Analysis including all patients with urticaria showed a positive correlation between IL-6 concentration and white blood count (WBC) (r = 0.21, *p* = 0.03), neutrophil-to-lymphocyte ratio (NLR) (r = 0.2, *p* = 0.04) and neutrophil count (r = 0.2, *p* < 0.05) and IL-17A concentration and CRP (r = 0.35, *p* < 0.001). In AU and CU patients, there was a correlation between IL-6 and CRP levels (r = 0.38, *p* = 0.03; r = 0.6, *p* < 0.001, respectively). Duration of hospitalization correlated with IL-6 levels in AU patients (r = 0.59, *p* < 0.001). Additionally, for CU, there was a correlation between IL-17A levels and D-dimers (r =  0.38, *p* =  0.03).

There were no significant correlations between the concentrations of the cytokines and chemokines studied in relation to age, BMI of the patients, and urticaria activity as determined by TSS. The comparison of cytokine and chemokine concentrations in the study and control groups is presented in Table 2.

**Table 2:** Comparison of the concentration of selected cytokines and chemokines in subgroups

Group/Parameter		Urticaria (*n* = 64)		Control group (n = 40)
IL-6 (pg/ml)		AU (*n* = 32)	13.27 (10.25–15.5)*	7.42 (6.21–8.45)
		CU (*n* = 32)	13.91 (11.32–15.71)*	
		Mild CU (*n* = 14)	13.82 (11.4–14.53) *	
		Moderate CU (*n* = 8)	12.72 (11.09–14.43) *	
		Severe CU (*n* = 10)	15.71 (11.35–17.82) *	
IL-17A (pg/ml)		AU (*n* = 32)	41.5 (38.35–44.15) *	27.13 (20.37–36.45)
		CU (*n* = 32)	41.4 (38.55–48.25) *	
		Mild CU (*n* = 14)	42.95 (39.6–47.6) *	
		Moderate CU (*n* = 8)	40.5 (34.3–45.55) *	
		Severe CU (*n* = 10)	41.45 (38.4–50.2) *	
IL-18 (pg/ml)		AU (*n* = 32)	114.2 (100.45–125.5) *	133.25 (122.95–142.15)
		CU (*n* = 32)	115.8 (102.5–129.5) *	
		Mild CU (*n* = 14)	115.05 (101.5–130.1) *	
		Moderate CU (*n* = 8)	111.9 (101.9–123.35)*	
		Severe CU (*n* = 10)	123.2 (104.7–132.1)*	
IL-23 (pg/ml)		AU (*n* = 32)	376.5 (308–434.5) *	603.0 (527.5–674.0)
		CU (*n* = 32)	361.5 (289.0–462.0) *	
		Mild CU (*n* = 14)	307.0 (289.0–364.0) *	
		Moderate CU (*n* = 8)	459.0 (411.0–506.5) *	
		Severe CU (*n* = 10)	365.0 (275.0–463.0) *	
RANTES (pg/ml)		AU (*n* = 32)	3791.0 (2896.5–4516.5) *	5822.0 (5337.5–6261)
		CU (*n* = 32)	3735.5 (3372.5–4676.5) *	
		Mild CU (*n* = 14)	4190.0 (3381.0–5127.0) */ **	
		Moderate CU (*n* = 8)	3140.0 (2806.5–3596.0) */***	
		Severe CU (*n* = 10)	3871.5 (3725.0–4271.0) *	
IP-10 (pg/ml)		AU (*n* = 32)	48.25 (41.95–51.2) *	73.25 (65.85–85.6)
		CU (*n* = 32)	48.0 (39.95–50.35) *	
		Mild CU (*n* = 14)	49.3 (43.2–50.6) *	
		Moderate CU (*n* = 8)	45.3 (38.1–49.15) *	
		Severe CU (*n* = 10)	47.45 (39.7–50.2) *	

Data presented as median (Q_25_ - Q_75_)

*AU* acute urticaria, *CU* chronic urticaria, *RANTES* Regulated upon Activation Normal T-cell Expressed and Secreted, IP-10 - IFN-γ-inducible protein-10

*p < 0.05 in comparison to the control group

**p < 0.05 children with mild CU vs moderate CU

***p < 0.05 children with moderate CU vs severe CU

Logistic regression analysis was performed to determine potential factors associated with the acute phase of urticaria. The model was built for the AU and CU patient groups combined. It was found that urticaria was associated with higher concentrations of IL-6 and IL-17A and lower values of IL-18, IL-23, RANTES and IP-10. The parameter with the highest odds ratio was IL-6 (OR 3.35; *p* < 0.001) - detailed data are presented in Table 3.

**Table 3:** Logistic regression analysis of the relationship between selected factors and the acute phase of urticaria

Parameter	OR	−95% CI	+ 95% CI	p
IL-6	3.35	1.98	5.66	< 0.001
IL-17A	1.24	1.14	1.35	< 0.001
IL-18	0.94	0.91	0.97	< 0.001
IL-23	0.97	0.96	0.98	< 0.001
RANTES	0.998	0.997	0.999	< 0.001
IP-10	0.76	0.68	0.85	< 0.001

*OR* odds ratio, *95% CI* lower and upper limits of the 95% confidence interval for the odds ratio, *RANTES* Regulated upon Activation Normal T-cell Expressed and Secreted, *IP-10* IFN-γ-inducible protein-10

**Fig. 1:**
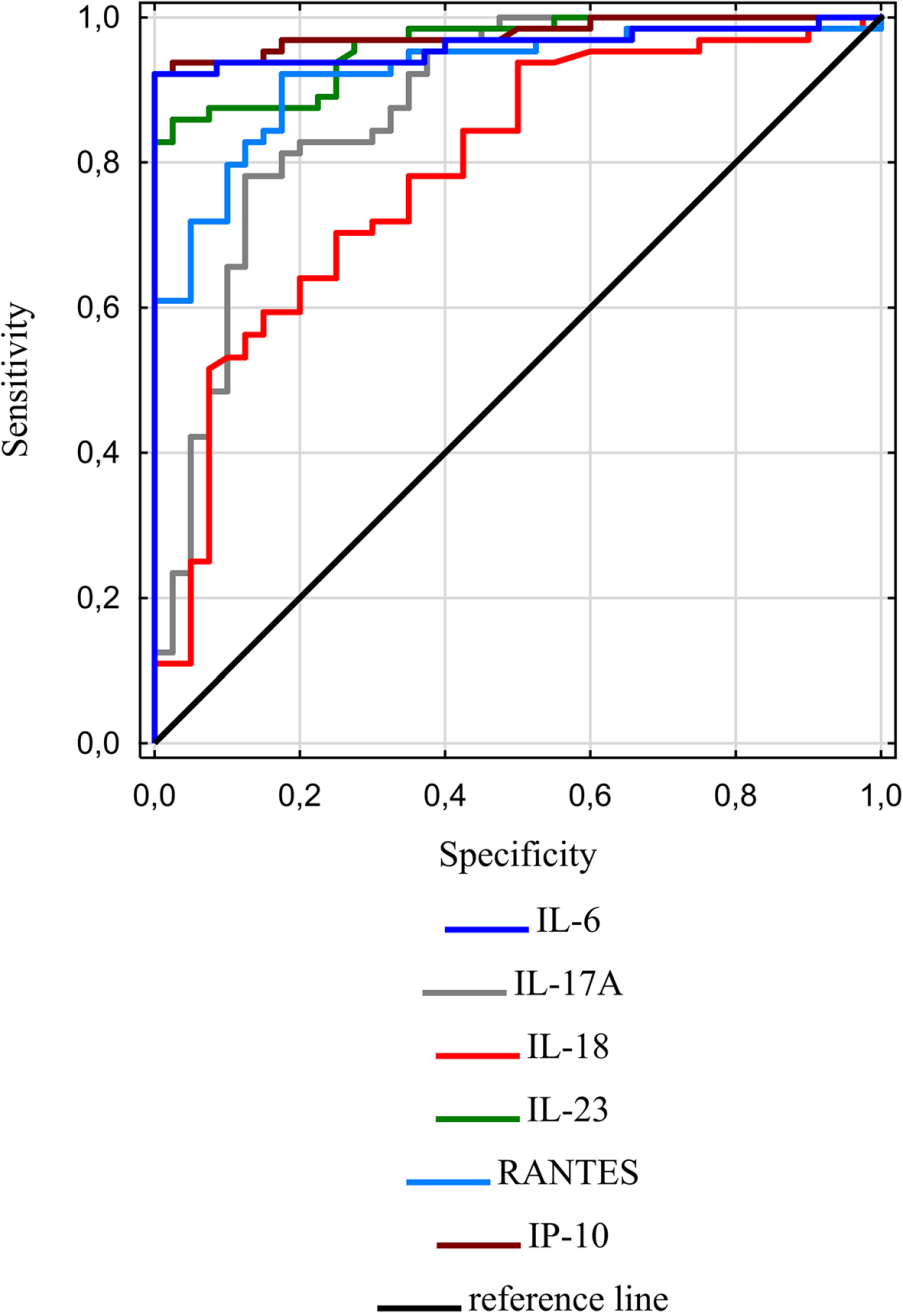
Comparison of receiver operating characteristic (ROC) curves of IL-6, IL-17A, IL-18, IL-23, RANTES and IP-10 for predicting the acute phase of urticaria

On the basis of ROC curve analysis, the largest area under the curve (AUC) was found for IP-10 (AUC = 0.98; p < 0.001), for which the optimal cut-off value determined by the Youden index was 55.10. The sensitivity of IP-10 for diagnosis of the acute phase of urticaria was calculated at 92% and specificity at 100%. Similar sensitivity and specificity (92 and 100%, respectively, for a cut-off point of 9.85) were demonstrated for IL-6, whose AUC was 0.96 (*p* < 0.001). The results are shown in Table 4, the comparison of ROC curves is provided in Fig. 1.

**Table 4:** Receiver operating characteristic (ROC) curves analysis of selected parameters as predictors of the acute phase of urticaria

Parameter	AUC	95% AUC CI	Cut-off	Sensitivity %	Specificity %	Youden index	p
IP-10	0.98	0.95–1.00	55.10	92	100	0.9219	< 0.001
IL-6	0.96	0.92–1.00	9.85	92	100	0.9219	< 0.001
IL-23	0.96	0.93–0.99	481.00	86	98	0.8344	< 0.001
RANTES	0.92	0.87–0.97	5241.00	92	83	0.7469	< 0.001
IL-17A	0.88	0.81–0.95	38.20	78	88	0.6563	< 0.001
IL-18	0.79	0.70–0.88	123.90	70	75	0.4531	< 0.001

*AUC* area under curve, *95% AUC CI* lower and upper limits of the 95% confidence interval for AUC, *RANTES* Regulated upon Activation Normal T-cell Expressed and Secreted, *IP-10* IFN-γ-inducible protein-10

## Discussion

In our study, similar to previous reports, we noted higher IL-6 levels in AU [5] and CU [6] patients. This increase was previously found in patients with antihistamine-resistant urticaria [9] and in severe CU compared to patients with mild CU [16]. We did not confirm the correlation of IL-6 levels with disease activity; however, this correlation has been documented in previous work [16–18]. We noted a correlation between IL-6 levels and CRP for AU and CU and IL-6 levels and duration of hospitalization in AU. In addition, we confirmed that IL-6 concentration correlates with WBC, NLR, and neutrophil count. ROC analysis showed that IL-6 has high sensitivity and specificity as a parameter associated with the acute phase of urticaria.

IL-6 is an acute phase protein closely related to the production of CRP and fibrinogen [3], whose levels are elevated during urticaria exacerbations [6, 19]. It stimulates the production of tissue factor linking coagulation and inflammation [6]. The expression of IL-6 is dependent on many factors such as coexisting chronic diseases, stress factors, trauma or infections [9], its concentration is also subject to circadian fluctuations [17].

We demonstrated significantly higher levels of IL-17A in patients with AU and CU compared to CG. In association with the acute phase of urticaria, this index with a relatively high specificity showed a lower sensitivity than most of the cytokines tested (88 and 78%, respectively, cut-off point 38.2). So far, most reports, like our work have shown higher IL-17 levels in CU patients [3, 9] compared to CG. Additionally, this increase was found in patients with CU and a positive autologous serum skin test (ASST) [20]. Atwa et al. reported a correlation between IL-17 levels and urticaria severity [21], which was not confirmed by Chen et al. [4]. Contrary to previous work [7], we confirmed a positive correlation between IL-17A levels and CRP in all patients with urticaria. We also found a correlation between IL-17A and D-dimer levels in patients with CU.

IL-17A stimulates the production of proinflammatory cytokines like IL-1 and IL-6 and adhesion molecules [7]. It plays a role in recruitment of neutrophils present in urticarial infiltrate [8, 22]. Together with IL-23, it is involved in the development of autoimmune mechanisms, which is of particular importance for CU [7, 21]. IL-23 directs the early immune response and influences T-cell differentiation toward Th17 [8].

Our analysis showed significantly lower IL-23 levels in AU and CU patients with respect to CG. Different results were obtained in several papers on CU [3, 4, 20], where additionally a correlation between IL-23 levels and CU severity was shown [4]. On the other hand, Degirmenci et al. reported lower levels of IL-17 and IL-23 in patients with CU, which was explained by the effect of cytokine consumption during the ongoing inflammatory process [8]. This parameter showed relatively high sensitivity and specificity for diagnosis of the acute phase of urticaria (86 and 98%, respectively; cut-off point 481.0).

In this study, we found significantly lower IL-18 levels in AU and CU patients compared with CG. ROC curve analysis showed that IL-18 as a parameter associated with the acute phase of urticaria had the lowest sensitivity and specificity among the parameters studied (70 and 75%, respectively; cut-off point 123.9). Previous studies have shown elevated IL-18 levels in children with a single episode of AU compared to patients with recurrent urticaria and healthy volunteers [11]. Two papers reported higher IL-18 levels in adults with CU [23, 24], while other studies did not confirm such an increase or the relationship between IL-18 levels and positive ASST and urticaria activity [3, 25].

IL-18 is responsible for the regulation of Th1, Th2, and Th17 responses, stimulates mast cell degranulation, and affects the recruitment of neutrophils and eosinophils to the site of inflammation [11, 23]. It is involved in the development of many diseases including CU and autoinflammatory syndromes (cryopyrin-associated periodic syndrome - CAPS and Schnitzler syndrome) [10].

Our analysis showed lower serum levels of RANTES and IP-10 in patients with AU and CU. In the ROC curve evaluation, IP-10 proved to be a statistically significant factor associated with the acute phase of urticaria, but no correlation with disease activity was demonstrated.

The relationship between RANTES and IP-10 has been investigated in a small number of papers finding higher levels in CU patients [15, 26], but the data are inconclusive. Puxeddu et al. showed no correlation of RANTES with disease activity [15], while Caproni et al. found no difference in IP-10 concentrations in CU patients regardless of ASST score [27].

RANTES induces histamine release from basophils and promotes differentiation and activation of mast cell progenitor cells [3, 15]. Both IP-10 and RANTES have been shown to play an important role in Th1 lymphocyte recruitment [14, 15]. IL-17 decreases lymphocyte recruitment by reducing RANTES production [28], which could indirectly explain the higher IL-17 concentrations we found with concomitantly lower RANTES levels in patients with urticaria.

The presented study is characterized by several limitations. These include the single-center, retrospective nature of the study, the limited size of the groups, and the lack of prospective determinations during the remission or convalescence phase. Urticaria activity in all patients was assessed according to the TSS scale, which does not provide more information about the patients’ quality of life relevant to the overall evaluation. Cut-off points were self-selected to qualify for a group with specific urticaria severity, which may affect the interpretation of the results. It should be noted that the development of acute and chronic urticaria depends on complex immune mechanisms. Despite the fact that in both groups we obtained similar results of cytokine and chemokine concentrations, the evaluation of immunological processes occurring in AU and CU requires further research.

## Conclusions

The cytokine serum levels in children with acute urticaria and exacerbation of chronic urticaria differs significantly compared with healthy children. Among the cytokines and chemokines studied, IL-6 and IP-10 seem to be useful in differentiating children with acute phase of urticaria and healthy ones. There is a need for further research to identify biomarkers predicting the course of urticaria.

## Data Availability

The data that support the findings of this study are available on request from the corresponding author.

## Abbreviations

APAACI: Asia Pacific Association of Allergy, Asthma and Clinical Immunology
AU: acute urticaria
AUC: area under curve
CG: control group
CIndU: chronic inducible urticaria
CSU: chronic spontaneous urticaria
CU: chronic urticaria
EAACI: European Academy of Allergology and Clinical Immunology
EDF; EuroGuiDerm: European Dermatology Forum
ELISA: Enzyme-linked immunosorbent assay
GA^2^LEN: Global Allergy and Asthma European Network
IFN-γ: interferon-γ
Ig: immunoglobulin
IL: interleukin
IP-10/CXCL10: interferon-γ-inducible protein-10
NLR: Neutrophil-to-lymphocyte ratio
NSAIDs: nonsteroidal anti-inflammatory drugs,
OR: odds ratio
PLR: Platelet-to-lymphocyte ratio
RANTES/CCL5: regulated on activation, normal T cell expressed and secreted
ROC: Receiver Operating Curve
Th: T-helper lymphocytes
TSS: Total Symptom Score
WBC: white blood count
